# Seasonal Composition and Structure of Methane-Cycling Communities in Alpine Lake Sediments of the Rila Mountains

**DOI:** 10.3390/microorganisms14061180

**Published:** 2026-05-23

**Authors:** Boyanka Angelova, Silvena Boteva, Anelia Kenarova

**Affiliations:** Department of Ecology and Environmental Protection, Faculty of Biology, Sofia University “St. Kliment Ohridski”, 8 Dragan Tzankov Blvd., 1164 Sofia, Bulgaria; b_angelova@biofac.uni-sofia.bg (B.A.); kenarova@biofac.uni-sofia.bg (A.K.)

**Keywords:** CH_4_, Rila Mountains, alpine lakes, methanogenic archaea, methanotrophic bacteria, co-occurrence network, next-generation sequencing

## Abstract

The global methane budget is largely driven by biogenic sources, many of which remain insufficiently characterized. Here, we investigated the community composition and seasonal dynamics of methanogenic and methanotrophic assemblages to elucidate the key contributors to methane cycling and the environmental factors shaping these processes in lake sediments of the Rila Mountains (Bulgaria). Methanogenic communities are primarily composed of *Methanothrix*, *Methanosarcina*, *Methanobacterium* and *Methanoregula* with summer peaks in *Methanothrix* and *Methanoregula*, and cold-season proliferation of *Methanobacterium*. Methanotrophic communities are dominated by representatives of the Pseudomonadota, including *Crenothrix*, *Methylobacter*, and *Methylocystis* with summer maxima observed for *Crenothrix* and *Methylobacter*. *Methanosarcina* and *Methylocystis* showed relatively stable abundances throughout the ice-free season. Ordination and correlation analyses revealed that temperature, pH, and carbon (organic and inorganic) concentration and lability emerged as the environmental drivers influencing on microbial communities, with seasonally variable effects on methane-cycling microorganisms. These findings provide a foundation for future research on methane cycling in alpine lake ecosystems of the Rila Mountains and contribute to improving predictions of methane emissions under changing climatic conditions.

## 1. Introduction

Methane (CH_4_) is a greenhouse gas with a global warming potential substantially exceeding that of CO_2_, rendering its production and consumption in natural ecosystems a critical component of the global carbon cycle [1,2]. According to Rosentreter et al. [3], freshwater wetlands (138–165 Tg yr^−1^) and lakes (23–142 Tg yr^−1^) constitute the largest aquatic sources of atmospheric CH_4_ emissions.

CH_4_ fluxes in natural environments are governed by the balance between methanogenic (MGs) and methanotrophic (MTs) microorganisms, which differ fundamentally in phylogenetic domain (Archaea vs. Bacteria) and ecological adaptations. MGs comprise a phylogenetically diverse group of anaerobic archaea that produce CH_4_ via three principal metabolic pathways: (i) the hydrogenotrophic pathway, in which CO_2_ is reduced with H_2_ to form CH_4_ and H_2_O; (ii) the acetoclastic pathway, involving the cleavage of acetate into CH_4_ and CO_2_; and (iii) the methylotrophic pathway, in which methanol and other methylated compounds are converted to CH_4_ [1]. Globally, methanogenesis is dominated by the acetoclastic and hydrogenotrophic pathways, contributing approximately 70% and 30% of total CH_4_ production, respectively [4,5]. Key methanogenic taxa include members of the genera *Methanoregula*, *Methanolinea*, *Methanobacterium*, and *Methanosaeta* [6].

In contrast, MTs mitigate CH_4_ emissions by oxidizing CH_4_ to CO_2_. These organisms are predominantly aerobic bacteria that utilize methane monooxygenase enzymes to initiate CH_4_ oxidation [7,8,9]. Most characterized MTs belong to the phyla Pseudomonadota and Verrucomicrobiota [10]. Pseudomonadota MTs are broadly divided into type I and type II groups, corresponding to the classes Gammaproteobacteria and Alphaproteobacteria, respectively, which differ in both substrate affinity and metabolic traits [11]. In freshwater environments, Gammaproteobacteria are commonly represented by the genera *Methylobacter*, *Methylomonas*, *Methylosarcina*, and *Methylomicrobium* [12,13,14], whereas Alphaproteobacteria are typically dominated by *Methylocystis*, *Methylocella*, and *Methylocapsa* [15,16,17].

In addition to aerobic methanotrophy, anaerobic methane oxidation (AOM) constitutes a major pathway for CH_4_ consumption in oxygen-depleted environments. Anaerobic MTs occur within both the archaeal phylum Halobacteriota and the bacterial phylum Methylomirabilota [18,19,20]. They typically form syntrophic consortia with partner microorganisms, where CH_4_ oxidation is coupled to the reduction in alternative electron acceptors, including sulfate, manganese, nitrate, nitrite, and iron. By utilizing these electron acceptors, AOM not only mitigates CH_4_ fluxes but also drives the transformation and mobility of key elements in aquatic ecosystems, thereby exerting broad control over biogeochemical cycling and ecosystem chemical balance [18,19,20,21].

The activity and distribution of CH_4_-cycling microorganisms are strongly influenced by environmental factors, including substrate availability, temperature, and redox potential [2,22]. For instance, organic matter concentration and its lability directly regulate methanogenesis by controlling substrate supply, whereas their effect on methanotrophy is largely indirect, mediated through oxygen depletion during mineralization or the production of alternative electron acceptors under anoxic conditions [23,24]. Redox stratification in lakes governs the spatial separation of CH_4_ production and consumption, with the intensity and vertical positioning of redox gradients strongly determining the fraction of CH_4_ that is oxidized before reaching the atmosphere [1]. Furthermore, temperature modulates CH_4_ dynamics. It affects methanogenesis and methanotrophy both directly, by altering microbial activity (MGs and MTs) and community composition (mainly MTs), and indirectly, by regulating substrate availability [25,26,27]. For instance, acetoclastic methanogenesis predominates at low temperatures, whereas both acetoclastic and hydrogenotrophic pathways contribute substantially to CH_4_ production under moderate temperatures [25,28].

Despite growing recognition of freshwater systems as dynamic contributors to global CH_4_ emissions [29,30], the distribution and seasonal dynamics of MGs and MTs in alpine lakes remain poorly understood. Only a limited number of studies, primarily focused on Tibetan Plateau lakes [2,31,32,33,34] characterized CH_4_-cycling communities and their functional traits. Comparable data are lacking for the Seven Rila Lakes, situated in the alpine zone of the Rila Mountains, Bulgaria.

The Rila Mountains represent one of the highest and most prominent mountain massifs in Southeastern Europe. It is located in the southwestern part of Bulgaria–southeastern region of the Balkan Peninsula. Unlike the European Alps, which are characterized by extensive glaciation, large interconnected high-altitude systems, and relatively uniform alpine climatic zones, the Rila Mountains form a more isolated and compact high-elevation system. This isolation contributes to a higher degree of environmental heterogeneity and reduced ecological connectivity between habitats. Compared to many alpine regions worldwide, such as the Alps, the Carpathians, or the Himalayas, the Rila Mountains experience a more pronounced continental climate with greater seasonal temperature amplitude [35]. Geologically, Rila is dominated primarily by crystalline rocks such as granite and metamorphic formations [36], which often result in low nutrient availability and weak buffering capacity in surface waters. This contrasts with many alpine regions where sedimentary or mixed lithologies can contribute higher nutrient inputs and greater geochemical diversity.

The cirques at the high elevation zone of the mountains contain over 140 glacial lakes, and their location is closely linked to the snowline during the last glacial period (2100–2500 m a.s.l.) [37]. These lake ecosystems are relatively small, oligotrophic to mesotrophic, and spatially isolated. In comparison to more extensively studied alpine systems in Central Europe and Asia, the Rila Mountains remain understudied, particularly in terms of microbial diversity and biogeochemical cycling, making them an important but still underrepresented region in global alpine ecology research. In this context, we hypothesize that the distinctive environmental conditions of the Rila Mountains may exert local specific influence on lake microbiota, as reflected in the composition of MG and MT assemblages, and may thereby shape local biogeochemical processes.

In this study, four key questions were addressed: (i) What is the composition and structure of CH_4_-cycling communities in the lakes of the alpine zone of the Rila Mountains?; (ii) How does the composition of methanogenic and methanotrophic communities vary seasonally?; (iii) Which metabolic pathways dominate in CH_4_ cycling?; (iv) What are the primary environmental factors influencing on CH_4_-cycling communities? Answering these questions is critical for assessing the microbial communities of the Rila lakes, as well as understanding how functional guilds within CH_4_-cycling assemblages respond to environmental changes.

## 2. Materials and Methods

### 2.1. Study Sites and Sampling Procedure

**Figure 1:**
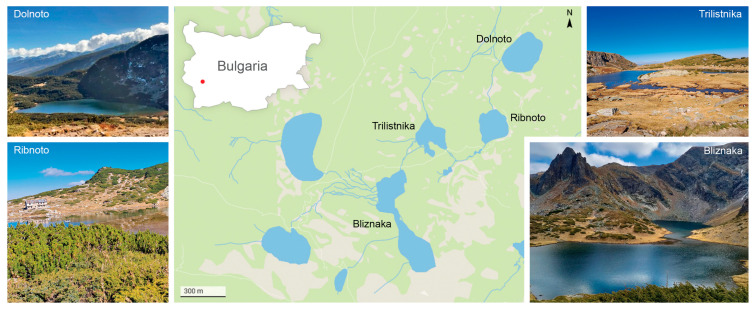
Map of the Seven Rila Lakes, northwest Rila Mountains, Bulgaria with the studied Bliznaka (42°12′02″ N, 23°18′57″ E), Trilistnika (42°12′22.0″N 23°19′06.0″E), Ribnoto (42°12′24″ N, 23°19′24″ E) and Dolnoto (42°12′39″ N, 23°19′32″ E) lakes. The map was created in Datawrapper [39].

The Seven Rila Lakes (Figure 1) are located within the Rila National Park and this region is included in the Natura 2000 network. The landscape is characterized by a distinct alpine morphology formed by seven stepped glacial cirques carved during the Würm glaciation period [35]. Land cover is predominantly composed of acidophilic grasslands (~80%) and shrub communities [38]. Located within the alpine and subalpine zones, the studied lake catchments span an elevation range from 2095 to 2243 m a.s.l. and catchment areas ranging from 205 ha (Bliznaka) to 281 ha (Dolnoto).

Hydrologically, the lakes are typically ice-covered from December to March, consistent with the prolonged sub-zero conditions of the high-mountain climate. Maximum water levels generally occur in May–June, driven by snowmelt runoff. Lake depth varies considerably among basins, with maximum depth of 27.5 m (Bliznaka), 11 m (Dolnoto), 6.5 m (Trilistnika), and 2.5 m (Ribnoto) [35].

Mean surface water temperatures across the studied lakes during June–October (2004–2017) ranged from 11.8 °C to 14.5 °C, while mean air temperatures at that time varied between 14.1 °C and 15.9 °C [35].

Sediment sampling campaigns were conducted during the ice-free periods of June, August, and October 2024, formally named ‘seasons’. The three sampling months were selected to represent the onset, peak, and termination of the lake vegetation growing season. To assess the complete microbial diversity within each lake’s littoral zone, upper-layer sediments (0–15 cm) were collected from two randomly selected 1 m × 1 m plots, each comprising three subsampling points. Subsamples from each plot were mixed in aliquots to generate representative samples (total of 24 composite samples) for subsequent microbial taxonomic analysis. All samples were collected in sterile containers, transported to the laboratory at 4 °C, and stored at −20 °C until DNA extraction.

### 2.2. Environmental Parameters

Sediment temperature (T, °C) was measured in situ, whereas all other sediment parameters were measured under laboratory conditions following sediment centrifugation and filtration of the supernatant (porewater), as described in Angelova et al. [40]. Porewaters were analyzed for pH, dissolved carbon (DC, mg L^−1^), dissolved inorganic carbon (DIC, mg L^−1^), dissolved organic carbon (DOC, mg L^−1^), and dissolved organic nitrogen (DON, mg L^−1^). In addition, the optical properties of DOC were characterized using SUVA_254_, E_2_/E_3_, and E_2_/E_4_ indices [40].

### 2.3. Molecular Analysis

#### 2.3.1. DNA Extraction

Total DNA was extracted from 24 sediment samples (250 mg per sample) using the Quick-DNA™ Fecal/Soil Microbe MiniPrep Kit (Zymo Research Corp., Irvine, CA, USA), following the manufacturer’s instructions. DNA concentration and purity (A260/280 ratio) were assessed using a micro-volume UV/Vis spectrophotometer (INNO, LTEK Co., Gyeonggi-do, Seongnam, Republic of Korea), and DNA integrity was evaluated by electrophoresis on a 1% agarose gel. The eluted DNA samples were stored at −20 °C until they were shipped and sequenced.

#### 2.3.2. Next-Generation Sequencing

Next-generation sequencing (NGS) of the extracted DNA was carried out by Novogene Co., Ltd. (Cambridge, UK) using the Illumina HiSeq platform, which generates approximately 30,000 tags per sample. For microbial identification, the V3–V4 hypervariable region of the 16S rRNA gene was amplified using the primer pair 341F/806R. Sequencing of sediment samples from Dol Lake in June and Tri Lake in August failed, resulting in a total of 22 datasets (7 samples both for June and August and 8 samples for October) for microbial taxa profiling. Primary bioinformatic analyses were also conducted by Novogene Co., Ltd. (UK).

#### 2.3.3. Data Processing and Analysis of Operational Taxonomic Units (OTUs)

Raw sequencing data generated by the NGS platform were initially demultiplexed and quality-trimmed. First, adapter sequences were trimmed and short reads (<20 bp) were removed using Cutadapt (v. 3.3). Paired-end reads were merged using FLASH software (v. 1.2.7) [41], with a minimum overlap of 10 bp and a maximum mismatch density of 0.2. Quality filtering on the raw tags was performed using the FASTP (v. 0.23.1) software to obtain high-quality clean tags following the criteria described by Bokulich et al. [42] and Caporaso et al. [43], resulting in datasets with average Q20 values above 99%. Chimeric sequences were identified and removed using the UCHIME algorithm implemented in VSEARCH (v. 2.16.0) [44] against the SILVA database (16S/18S). The resulting sequences were clustered into Operational Taxonomic Units (OTUs) using the UPARSE (v.7.0. 1001) algorithm at a 97% similarity threshold (-otu_radius_pct 3) [45].

To account for variations in sequencing depth (averaging ~30,000 reads/sample) and enable downstream comparisons, the OTU table was normalized (rarefied) to a consistent depth based on the sample with the lowest number of sequences. The adequacy of this sequencing depth was confirmed by calculating Good’s coverage index. Representative sequences for each OTU were taxonomically assigned by comparison with the SILVA SSU rRNA database (v. 138) using Mothur software (v. 1.48.0) [46,47]. Sequences identified as mitochondrion, chloroplast or unidentified 16S rDNA were removed from the datasets. The sequencing data have been deposited in the NCBI Sequence Read Archive (SRA) under the BioProject accession number PRJNA1398596.

To achieve a comprehensive characterization of CH_4_-cycling taxa, we systematically screened taxonomic assignments to identify well-established methanogenic and methanotrophic lineages within the broader archaeal and bacterial communities. This selection was guided by literature-derived evidence and validated against authoritative nomenclature resources, including the List of Prokaryotic names with Standing in Nomenclature (LPSN) [48], the National Center for Biotechnology Information taxonomy database (NCBI) [49], and the International Code of Nomenclature of Prokaryotes (ICNP) [50,51].

### 2.4. Statistical Analysis

Microbial statistical analyses were performed with MicrobiomeAnalyst software (v. 2.0) [52] and the significance level applied for all statistical tests was 5% (*p* < 0.05). Statistical significance was inferred using the Mann–Whitney/Kruskal–Wallis method for alpha diversity and classical univariate comparison analysis, whereas PERMANOVA was used to test the statistical significance of dissimilarity measures. Alpha-diversity [53] was calculated based on the sequence similarity at the level of 97%. The richness was calculated as the amount of unique OTUs found in each sample and presented as observed OTUs. The count of unobserved species based on low-abundance OTUs was presented as ACE and Chao1 indices [54,55,56]. Shannon index was calculated to measure both the richness and dominance within individual samples [53]. Beta-diversity (the distance and dissimilarities between microbial communities) was determined based on the Bray–Curtis dissimilarity index and the distances were visualized by principal coordinate analysis (PCoA) [57].

Redundancy analysis (RDA) was performed to assess relationships among microbial communities at a family level, and relationships between community composition and measured environmental variables. The statistical significance of the ordination models and canonical axes was evaluated using permutation tests (999 permutations). The proportion of explained variance was used to quantify the contribution of environmental variables to community variation. Ordination axes were interpreted based on correlations between environmental variables and canonical axes (|≥0.3|), as indicated by vector direction and magnitude in the ordination space. In addition, Pearson correlation analysis was conducted to further support the relationships identified by RDA. All analyses were performed in PAST (v. 4.03) at *p* ≤ 0.05 [58].

## 3. Results

### 3.1. Diversity and Richness of Methane Cycling Communities

**Figure 2:**
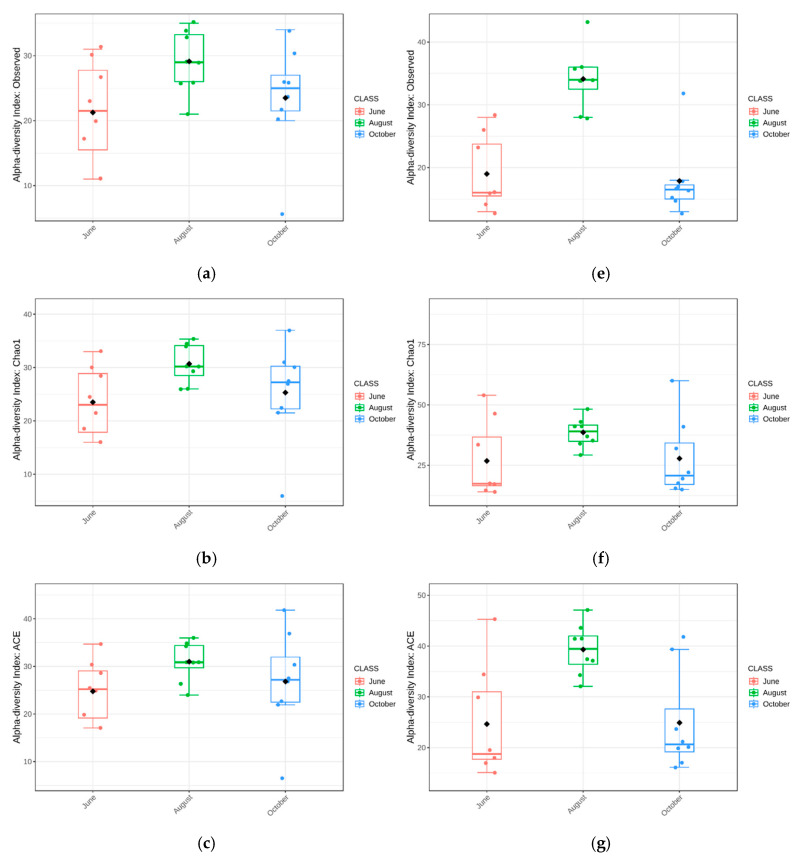

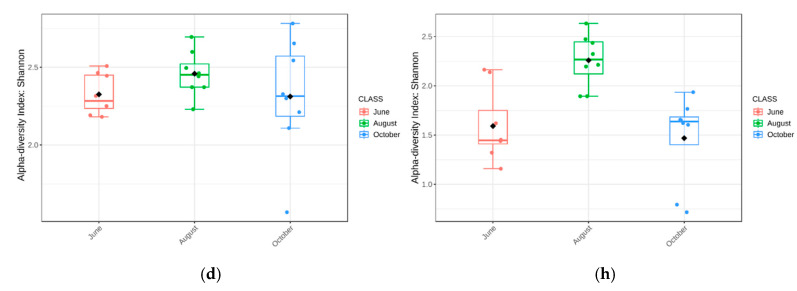
Indices of archaeal (**a**–**d**) and bacterial (**e**–**h**) richness, expressed as Observed OTUs (**a**,**e**), Chao1 (**b**,**f**) and ACE (**c**,**g**), as well as Shannon diversity (**d**,**h**), illustrating the overall structure of the CH_4_-cycling sediment microbiota.

Alpha-diversity metrics were applied to characterize the microbial CH_4_-cycling communities using three complementary measures: observed richness (number of OTUs), estimated richness (Chao1 and ACE indices), and community diversity (Shannon index) (Figure 2).

Chao1/ACE estimates of bacteria were significantly higher than Observed OTUs (F ≥ 4.2, *p* ≤ 0.049), reflecting the presence of low abundance OTUs. No significant differences among these metrics were detected for archaea (F ≥ 0.76, *p* ≤ 0.20).

Seasonal comparisons of alpha diversity indices indicated that both microbial richness (bacteria: 34 ± 4.8; archaea: 29 ± 4.8) and diversity (bacteria: 2.26 ± 0.26; archaea: 2.46 ± 0.14) peaked in August.

**Figure 3:**
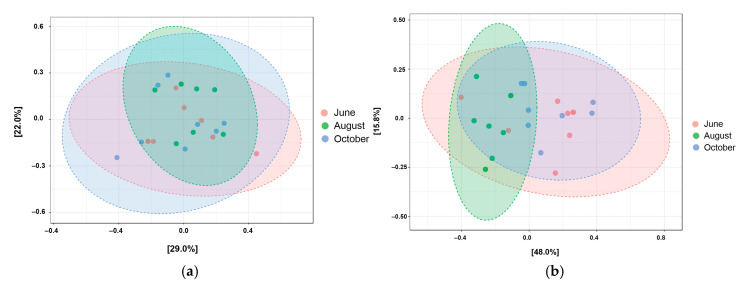
Principal coordinate analysis (PCoA) plots with 95% confidence ellipses of sediment microbiota based on Bray–Curtis dissimilarity index for archaeal (**a**) and bacterial (**b**) CH_4_-cycling assemblages in June (pink), August (green) and October (blue), with points representing the sampling plots.

Further, to explore the differences in archaeal and bacterial CH_4_-cycling communities between seasons (sampling months), beta-diversity was assessed using the Bray–Curtis index, and the resulting scores were visualized as PCoA plots (Figure 3a,b).

Seasonal differentiation of CH_4_-cycling communities was primarily structured along the first principal coordinate, which accounted for 29% and 48% of the total variation in archaeal and bacterial communities, respectively. For bacteria, the seasonal differences between June, August and October were statistically supported by PERMANOVA (n = 22; F = 3.72, R^2^ = 0.26, *p* = 0.002), despite partial overlap of the 95% confidence ellipses. In contrast, no significant seasonal effect on community structure was detected for archaea (n = 22; F = 0.578, R^2^ = 0.07, *p* = 0.71), indicating a high degree of similarity among seasons.

### 3.2. Community Composition of Methane Cycling Archaea

A total of 49 archaeal OTUs were affiliated with 3 phyla, comprising 8 identified methanogenic families (41 OTUs) and 9 genera (33 OTUs), as well as 1 methane oxidizing family (1 OTU) with 1 genus (Table 1).

**Figure 4:**
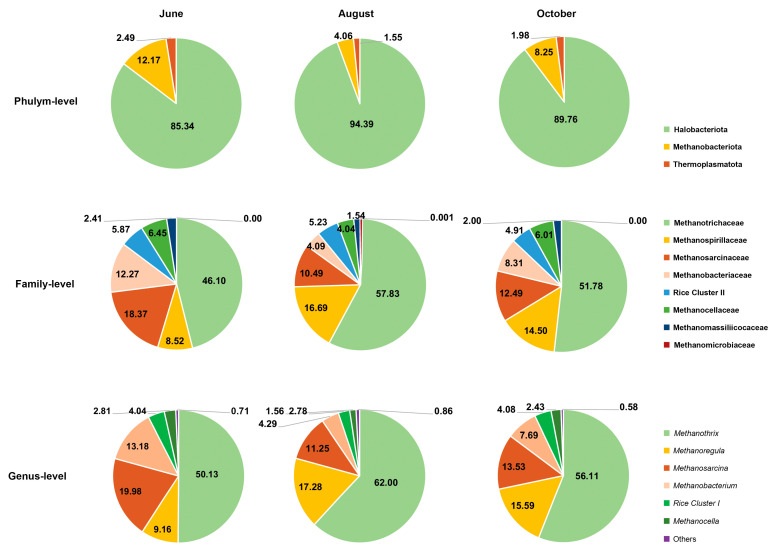
Relative abundances of methanogenic archaeal phyla, families and genera in sediments of Rila lakes across sampling months.

The identified MG phyla were Halobacteriota with a relative abundance of 85–94%, Methanobacteriota (4–12%) and Thermoplasmatota (1.6–2.5%). The relative abundances of the three detected phyla were generally stable throughout the sampling period, with minor shifts observed in August, characterized by an increase in Halobacteriota and a decrease in Methanobacteriota (Figure 4). Among the phylum Halobacteriota, CH_4_ oxidizing members of the family Methanoperedenaceae (genus Ca. *Methanoperedens*) were identified, and they accounted for less than 0.3% of the CH_4_-cycling archaeal community in Rila lakes.

Most of the identified MG families (Methanocellaceae, Methanomicrobiaceae, Methanospirillaceae, Rice Cluster II, Methanotrichaceae, and Methanosarcinaceae) belonged to the phylum Halobacteriota, whereas Methanobacteriaceae and Methanomassiliicoccaceae were affiliated with phyla Methanobacteriota and Thermoplasmatota, respectively. Methanotrichaceae was the most abundant family (46–58%), followed by Methanospirillaceae (8.5–17%) and Methanosarcinaceae (10.5–18%). The dominant genus was *Methanothrix* (formerly *Methanosaeta*; 50–62%; Methanotrichaceae), followed by *Methanosarcina* (11–20%; Methanosarcinaceae) and *Methanoregula* (9–17%; Methanospirillaceae).

Seasonal patterns at the family level revealed significant shifts in August relative to June, including a threefold (Methanospirillaceae) and a twofold (Methanotrichaceae) increase in OTU reads, corresponding to increases in the genera *Methanoregula* and *Methanothrix*, respectively. The abundances of Methanosarcinaceae, Methanobacteriaceae, and Methanocellaceae remained relatively stable between June and August, but increased markedly in October by 27–66%. This increase was primarily driven by the proliferation of the genera *Methanosarcina*, *Methanobacterium*, and *Methanocella*, respectively.

### 3.3. Community Composition of Methane Cycling Bacteria

A total of 82 bacterial OTUs of methanotrophs were affiliated with three phyla (Pseudomonadota, Methylomirabilota and Verrucomicrobiota), encompassing five identified families (82 OTUs) and 13 genera (31 OTUs) (Table 2).

**Figure 5:**
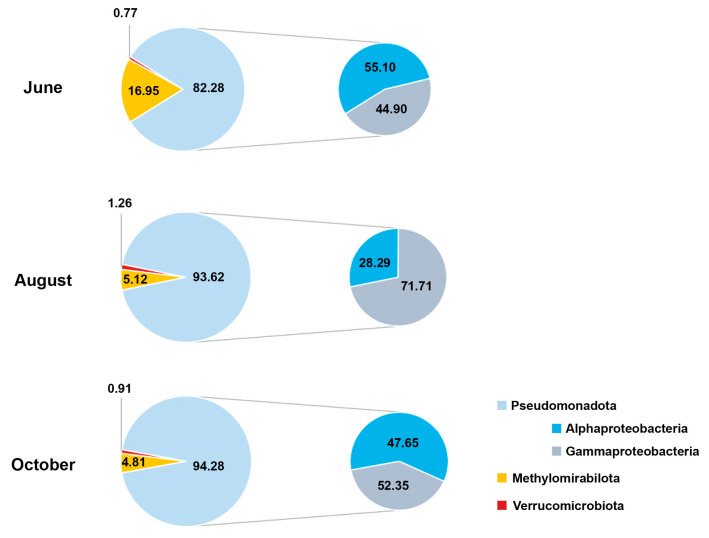
Relative abundances of methanotrophic bacterial phyla in sediments of Rila lakes across sampling months.

Pseudomonadota dominated, accounting for 82–94% of total bacterial community (Figure 5). Within this phylum, class-level representation varied with Alphaproteobacteria comprising 28–55% of the phylum abundance, and Gammaproteobacteria ranging from 45% to 72%. Methylomirabilota and Verrucomicrobiota accounted approximately 5–17% and 0.8–1.3%, respectively.

Seasonal dynamics across these phyla indicated a marked restructuring of the MT assemblages in August, reflected by a decrease in the relative abundance of Alphaproteobacteria and a concomitant increase in Gammaproteobacteria (Figure 5).

**Figure 6:**
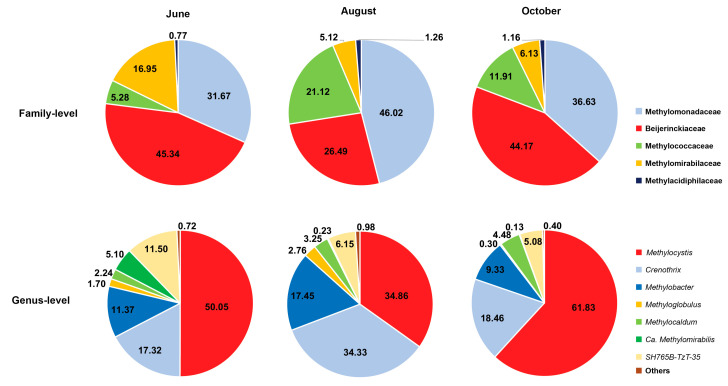
Relative abundances of methanotrophic bacterial families and genera in sediments of Rila lakes across sampling months.

The most abundant bacterial families (relative abundance > 10%) were Methylomonadaceae (38.4%), Beijerinckiaceae (38.3%), and Methylococcaceae (14.7%). On average, Methylomirabilaceae and Methylacidiphilaceae accounted for 7.9% and 1.1% of the bacterial community, respectively (Figure 6).

Seasonal variation in community structure showed an increase in the relative abundances of Gammaproteobacterial families Methylomonadaceae and Methylococcaceae from 37% in June to 67% in August. Although the absolute abundance of the Alphaproteobacterial family Beijerinckiaceae increased modestly from 1589 OTU counts in June to 2314 OTU reads in August, its relative contribution to the total bacterial community decreased markedly, from 45% to 26% at that time.

The identified CH_4_ oxidizing genera were classified as major (≥5%) or minor (<5%) according to their OTU counts. The major genera were *Methylocystis* (46.2%), *Crenothrix* (25.8%), *Methylobacter* (13.7%), and SH765B-TzT-35 (7.1%). Together with *Methylocaldum*, *Methyloglobulus*, and Ca. *Methylomirabilis*, these taxa collectively accounted for 99.2% of the total MT community structure. Seasonal dynamics among the major genera depicted an increased in the total relative abundance of *Crenothrix* and *Methylobacter* in August, while that of *Methylocystis* and SH765B-TzT-35 decreased for the same period. Notably, *Methylocystis* reached its peak abundance in October, accounting for 62% of the total MT community (Figure 6).

### 3.4. Relationships Among Methane Cycling Microorganisms. Communities and Environmental Factors

**Figure 7:**
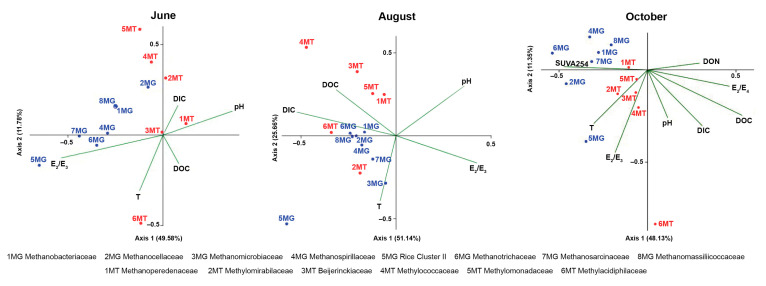
Redundancy analysis (RDA) illustrating the relationships between phylogenetic families of methane producers (MGs; blue) and methane oxidizers (MTs; red) in the sediments of Rila lakes and environmental variables: temperature (T), pH, dissolved organic (DOC) and inorganic (DIC) carbon, E_2_/E_3_ (DOC molecular weight), dissolved organic nitrogen (DON), source of DOC (E_2_/E_4_), and DOC aromaticity (SUVA_254_).

Redundancy analysis (RDA; Box–Cox transformed) was performed to examine the relationships between sediment MGs and MTs, as well as between microorganisms and environmental variables (pH, T, DOC, DIC, and E_2_/E_3_) across sampling months (June, August, and October) (Figure 7). DC, DON, E_2_/E_4_ and SUVA_254_ were excluded from the analysis due to multicollinearity. The values of sediment parameters and their fluctuation over time are published in Angelova et al. [40]. Family Methanomicrobiaceae (3MG) had no OTUs detected in June and October, and was excluded from the ordination analysis for these months.

The RDA models were statistically significant in June (adjusted R^2^ = 0.84, *p* = 0.047) and August (adjusted R^2^ = 0.72, *p* = 0.006), indicating that the selected environmental variables explained a substantial proportion of the variation in microbial community dispersion during these periods. In contrast, the October model was not significant (adjusted R^2^ = −0.15, *p* = 0.57), suggesting limited explanatory power of the selected variables under autumn conditions. To further evaluate potential environmental influences in October, a second ordination was performed including all measured environmental variables except DC. This expanded model was statistically significant (adjusted R^2^ = 0.93, *p* = 0.032), indicating that variables excluded from the initial analysis due to collinearity may collectively contribute to explaining microbial dispersion patterns during this period. However, the high explanatory power of the expanded October model should be interpreted cautiously due to the inclusion of collinear predictors.

The first two canonical axes explained 52% (June) to 77% (August) of the constrained variance. The RDA ordination plots revealed that both MG and MT communities clustered predominantly on the left side of the ordination space across all three sampling months, indicating close ecological relationships between these functional guilds. Additionally, MG communities had more compact clustering patterns, whereas MT communities were generally more dispersed, except in October when MT taxa formed a comparatively compact cluster.

Despite occupying similar regions of the ordination space, MG and MT assemblages were associated with different environmental vectors, indicating season-specific environmental influence on their abundance and community composition.

In June, MG communities were positively associated with the E_2_/E_3_ ratio, suggesting a relationship with DOC molecular weight, whereas most MT taxa showed a negative association with temperature (except Methylacidiphilaceae). Under summer conditions, MGs related positively to carbon (organic and inorganic) availability and negatively to pH and DOC molecular weight. The reversal in the relationship between MGs and E_2_/E_3_ from June to August may indicate that MGs respond differently to DOC quality during the onset versus the peak of the lake growing season, likely reflecting seasonal changes in substrate availability, microbial processing intensity, and sediment redox dynamics. DOC concentration and molecular characteristics in August also influenced MT abundance patterns, suggesting increasing coupling between methane production and oxidation processes in the peak of growing season. Although the October RDA model remained statistically significant and explained a high proportion of the total variance, the environmental vectors had relatively weak correlations with the ordination axes. Although vector directions at that time remained informative, the magnitude and independence of variable effects were likely influenced by shared variance among predictors, potentially reflecting environmental filtering rather than independent effects.

**Figure 8:**
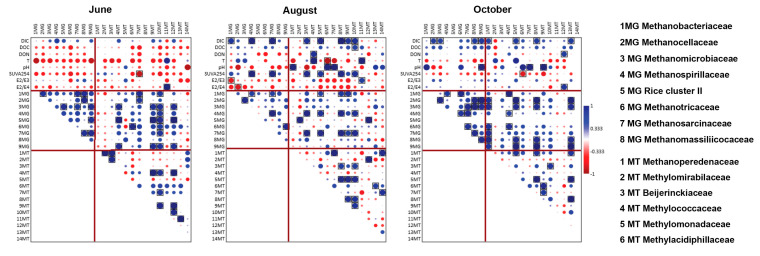
Relationships between families of methanogens (MG) and methanotrophs (MT), as well as between environmental variables and microorganisms in the Rila lakes in June, August and October (n = 14). Symbols: DIC—dissolved inorganic carbon; DOC—dissolved organic carbon; DON—dissolved organic nitrogen; SUVA_254_—aromaticity of DOC; E_2_/E_3_—molecular weight of DOC; E_2_/E_4_—source of DOC; T—temperature; and pH. Note: boxed correlations are significant (*p* < 0.05).

To further explore the relationships demonstrated by the RDA, Pearson correlation analysis was performed with more environmental variables (Figure 8).

Similarly, to the RDA, correlation analysis revealed pronounced seasonal restructuring of the sediment microbial communities from June to October. In June, the network between the functional guilds of MGs and MTs was comparatively sparse and characterized by limited cross-guild connectivity. Significant interactions among families were predominantly positive and largely concentrated within the MG guild. The negative correlations were infrequent, generally weak, and concentrated in the guild of MTs. By August, network complexity increased markedly, reflected in a higher number of relationships among taxa. Cross-guild correlations became more prevalent, indicating intensified co-occurrence patterns between functional guilds of MGs and MTs. The relationships within the MT guild also strengthened, shifting from negative and insignificant in June to positive and significant in August. In October, microbial cross-guild interactions remained complex, accompanied by a renewed increase in correlations within the MG guild.

Environmental influences on microbial community structuring largely corroborated the patterns observed in the RDA. Pearson correlation analysis revealed a progressive seasonal strengthening of environmental influence over CH_4_-cycling communities, with maximal relationships during the summer.

## 4. Discussion

### 4.1. Methanogenic Communities in Rila Lake Sediments—Main Characteristics

Methanogenic communities constitute a phylogenetically and metabolically diverse lineage within the domain Archaea, inhabiting not only extreme environments, as originally postulated, but also a wide spectrum of more common ecosystems, including lakes, wetlands, and soils [59]. The composition of sediment MG communities in the Rila sediments was dominated by *Methanothrix*, *Methanosarcina*, *Methanoregula*, and *Methanobacterium* (Table 1), consistent with previous reports for high alpine ecosystems [29,34,60]. The Rila lakes exhibited a relatively stable seasonal core community, with *Methanothrix* maintaining year-round dominance and reaching maximum abundance during summer (Figure 4). A similar summer peak was also observed for *Methanoregula*, whereas *Methanosarcina* and *Methanobacterium* peaked at the beginning (June) and end (October) of the growing season, periods characterized by lower sediment temperatures and reduced nutrient concentrations.

*Methanothrix*, *Methanosarcina*, *Methanoregula*, and *Methanobacterium* have likewise been reported as major contributors to MG communities in geographically distinct cold, high-altitude freshwater ecosystems, including the Qinghai Plateau [29,60], Tibetan Plateau [34], Yunnan Plateau [59], Chilean Altiplano [61], and the Canadian High Arctic [62]. Their recurrent occurrence across environmentally distinct ecosystems with contrasting hydrological and geochemical settings suggests that they constitute a conserved core methanogenic consortium adapted to cold freshwater habitats in alpine and polar regions.

The Rila MG dominants represent distinct functional pathways of CH_4_ production, including acetoclastic (*Methanothrix*), hydrogenotrophic (*Methanoregula* and *Methanobacterium*), and metabolically versatile (*Methanosarcina*) methanogenesis. Among these, the persistent dominance of *Methanothrix* indicates that acetoclastic methanogenesis is likely the principal pathway of CH_4_ production in Rila sediments. This pattern contrasts with observations from some alpine ecosystems, particularly the Tibetan Plateau, where hydrogenotrophic methanogenesis has been reported as the dominant pathway [29,34], but agrees with studies suggesting that acetoclastic methanogenesis generally predominates in aquatic ecosystems throughout the year [4,5,25,28]. Jones (1991) also stated that acetate is a key intermediate in the anaerobic food chain and estimated that up to two-thirds of biologically produced CH_4_ emitted to the atmosphere annually originated from the methyl group of acetate [63]. The predominance of alternative methanogenic pathways among high-altitude freshwater systems likely reflects differences in environmental and geochemical conditions, particularly trophic status, organic matter composition, acetate availability, H_2_/CO_2_ supply, temperature regime, and sediment redox dynamics.

In Rila sediments, hydrogenotrophic taxa were in relative lower abundance (4.3–17.3%) than *Methanothrix* and displayed marked seasonal niche partitioning. Specifically, *Methanoregula* was favored under warmer summer conditions, while *Methanobacterium* was relatively more abundant during colder periods at the beginning and end of the vegetation growing season. These seasonal dynamics suggest greater ecological specialization among hydrogenotrophic methanogens, with individual taxa responding to distinct environmental conditions. *Methanothrix* appears to function as a generalist capable of maintaining competitive dominance across seasonal fluctuations. Collectively, these findings refine previous observations by Wu et al. [25] and Levergne et al. [28], suggesting that CH_4_ production in the Rila lakes is predominantly driven by acetoclastic methanogenesis mediated by *Methanothrix*, whereas hydrogenotrophic methanogens contribute in a seasonally partitioned manner under both colder and warmer conditions. The persistent dominance of *Methanothrix* throughout the ice-free season, together with the comparatively high diversity of hydrogenotrophic taxa comprising distinct seasonal specialists, strongly supports this interpretation.

### 4.2. Methanotrophic Communities in Rila Lake Sediments—Main Characteristics

Methanogens in natural ecosystems commonly coexist with aerobic and anaerobic MTs, forming tightly coupled microbial consortia that regulate CH_4_ turnover through syntrophic interactions. These organisms act as an effective biological CH_4_ filter, substantially reducing CH_4_ emissions from anoxic environments [64,65]. Rila lake sediments harbored a taxonomically and functionally diverse methanotrophic community comprising both anaerobic and aerobic CH_4_ oxidizers. Anaerobic methanotrophy was represented by the low-abundance archaeal family *Methanoperedenaceae* (Table 1) and the more abundant bacterial family *Methylomirabilaceae* (Table 2), members of which are well known to couple methane oxidation to nitrate reduction and denitrification [66].

Aerobic methanotrophy was dominated by genera *Methylocystis*, *Crenothrix*, and *Methylobacter*, representing complementary ecological strategies for CH_4_ oxidation across dynamic redox gradients (Table 2). *Methylocystis*, a facultative type II methanotroph, is typically associated with low CH_4_ concentrations and greater metabolic flexibility [67,68,69,70], whereas *Methylobacter* and *Crenothrix* are key type I methanotrophs specialized in efficient CH_4_ oxidation under elevated methane fluxes [70,71,72,73]. Notably, *Crenothrix* is particularly adapted to fluctuating oxygen availability and methane-rich microenvironments, and may additionally couple CH_4_ oxidation to nitrate reduction, making it functionally important in methane–nitrogen transition zones [71,72,73].

The coexistence of all these taxa indicates a complementary methanotrophic network in Rila sediments, likely enhancing functional resilience under seasonally variable CH_4_ availability and redox conditions. This interpretation is supported by distinct seasonal dynamics of MTs, with *Crenothrix* and *Methylobacter* manifested pronounced increases in August.

The dominant methanotrophs detected in Rila sediments are consistent with patterns reported from other cold freshwater and alpine systems. Type I methanotrophs, particularly *Methylobacter* and *Crenothrix*, are frequently reported as dominant CH_4_ oxidizers in high-altitude lakes, where they constitute the principal CH_4_ sink in oxic–anoxic transition zones [2,71,73,74,75,76]. In contrast, *Methylocystis* are generally less abundant in alpine lakes and more commonly associated with adjacent soils and littoral terrestrial habitats [2]. Collectively, these results suggest that CH_4_ oxidation in the Rila lakes is mediated by a functionally versatile community combining dominant type I methanotrophs with facultative and anaerobic CH_4_ oxidizers, thereby maximizing CH_4_ consumption across heterogeneous sediment microhabitats.

### 4.3. Seasonal Relationships of Methane Cycling Microorganisms and Environmental Effects

Patterns of alpha- and beta-diversity (Figure 2, Figure 3, Figure 4, Figure 5 and Figure 6), as well as seasonal MG and MT profiles showed that the MG community was consistently more diverse and temporally stable than bacterial community of MTs. This pattern aligns with previous studies highlighting distinct ecological strategies across these phylogenetic domains, including enhanced environmental stress tolerance in archaea [77] and greater adaptive flexibility in bacteria [26,78]. Multivariate (RDA; Figure 7) and Pearson correlation (Figure 8) analyses revealed compact seasonal structuring of microbial communities, highlighting potential interaction networks and trophic connectivity between MGs and MTs. Associations among CH_4_ cycling microorganisms were predominantly positive, although their strength and network configuration varied across seasons. Overall, cross-guild interactions and intra-guild relationships within the MT guild were intensified during the peak and late growing season, whereas stronger correlations within the MG guild were characteristic of the colder periods at the beginning and end of the season.

DOC concentration and composition (E_2_/E_3_) were identified as the principal environmental factors shaping both functional guilds (Figure 7). The low abundance of highly recalcitrant DOC at the beginning of the growing season [40] appears to have been a key factor influencing MG community composition, abundance, and internal interactions. During this period, MGs likely depended primarily on the activity of psychrophilic fermentative microorganisms and their metabolic end products, including acetate, H_2_, and formate [79,80]. This interpretation is supported by the higher relative abundance of taxa associated with acetoclastic methanogenesis. Whereas lower spring temperature had no significant effect on MGs, it emerged as the primary environmental factor negatively associated with MT abundance (Figure 7). The comparatively higher temperature sensitivity of bacteria and lower sensitivity of archaea have been well documented [26,78,81,82]. For example, Conrad (2023) [82] reported that temperature strongly affects MG activity and CH_4_ production, while exerting only limited influence on MG community composition.

Higher summer temperatures, together with increased DOC availability and lability [40], driven by algal exudate production [83,84], created favorable conditions for the proliferation of both MGs and MTs. During this period, correlations between the two guilds increased substantially and became stronger, suggesting enhanced syntrophic coupling and more complete CH_4_ cycle. Similar seasonal convergence of environmental drivers shaping both MG and MT communities has been reported in other freshwater ecosystems, where elevated carbon lability and higher temperatures accelerate both CH_4_ production and oxidation [80,85,86]. Notably, partial decoupling within the MG guild was observed during summer, potentially reflecting increased substrate heterogeneity and niche differentiation among methanogens. The concurrent proliferation of acetoclastic, hydrogenotrophic, and metabolically versatile MGs (Table 1) further supports the coexistence of multiple methanogenic pathways and enhanced niche partitioning during this period.

The end of the growing season was characterized by lower temperatures and reduced carbon availability [40]. Despite these constraints, DOC was predominantly present as low-molecular-weight compounds, which likely sustained the numerous and strong cross-guild interactions observed during this period. Intra-guild interactions within MGs also strengthened, resembling the pattern observed at the beginning of the growing season. Collectively, these patterns suggest that the MG–MT system transitioned into an intermediate ecological state, potentially representing an early shift toward winter-like community organization.

These findings provide novel insight into the dynamic organization of CH_4_ cycling microbial communities by demonstrating that seasonal environmental forcing can restructure microbial assemblages from loosely connected to highly integrated interaction networks. Our results highlight a previously underappreciated sensitivity of CH_4_ cycling interactions to moderate climate-driven variation in temperature and carbon availability. Given that current understanding is derived largely from alpine lakes of the Tibetan Plateau, this study provides the first baseline characterization of CH_4_ cycling microbial communities in Balkan alpine lakes, and in this context expanding the existing biogeographical framework and underscoring the ecological significance of this understudied alpine region.

## 5. Conclusions

In this study, four lakes in Rila Mountain were selected as research objects to reveal the characteristics of CH_4_-cycling communities in sediments during cold (June and October) and warm (August) using Next-generation sequencing approach. The main conclusions are as follows:

(i) This study provides the first seasonal characterization of CH_4_-cycling microbial communities in alpine lakes of Rila Mountains, and contributes baseline data for underexplored Balkan alpine ecosystems. In this context, the study expands current understanding of microbial diversity and CH_4_ related processes in global alpine lake environments.
(ii) The dominant genera of CH_4_ cycling in Rila sediments are methanogenic *Methanothrix*, *Methanosarcina, Methanobacterium* and *Methanoregula* and methanotrophic *Crenothrix*, *Methylobacter* and *Methylocystis*;
(iii) The acetoclastic pathway in CH_4_ production is represented throughout the year by a high abundance of *Methanothrix*, whereas the hydrogenotrophic pathway is supported by *Methanobacterium* during the cold season and *Methanoregula* in the warmer summer;
(iv) Alpha diversity of both MG and MT communities increases during the summer, whereas beta diversity exhibits significant differences between seasons for MTs but not for MGs;
(v) Temperature, pH and DOC quantity and quality have significant impact on composition and diversity of CH_4_-cycling community. Seasonal changes in values of environmental factors drive a shift from decoupled in June to more tightly coupled microbial assemblages in August.

These findings underscore the sensitivity of CH_4_ cycling to environmental change and indicate that climate-driven shifts may rapidly restructure microbial communities and their functional capacity. To more fully resolve environmental controls on CH_4_ cycling, future work should extend beyond assessments of microbial abundance to include direct measurements of activity, enabling an integrated evaluation of the relationships among environmental parameters, community composition, and the activity of CH_4_-cycling microorganisms.

## Figures and Tables

**Table 1:** Methane cycling archaeal taxa and total OTU reads per genus in June (J), August (A) and October (O), in the littoral sediments of the Rila lakes.

Phylum	Family	Genera	OTU Reads per Genus		
			J	A	O
Methanobacteriota	Methanobacteriaceae	*Methanobacterium*	933	491	973
Halobacteriota	Methanocellaceae	*Methanocella*	199	178	308
		*Rice Cluster I*	285	318	516
	Methanospirillaceae	*Methanoregula*	648	1977	1973
		*Methanosphaerula*	4	1	0
		*Methanospirillum*	4	69	16
	Methanotrichaceae	*Methanothrix*	3551	7095	7102
	Methanosarcinaceae	*Methanosarcina*	1415	1287	1713
	Rice Cluster II		452	642	673
	Methanomicrobiaceae		0	10	0
	Methanoperedenaceae	Ca. *Methanoperedens*	26	20	5
Thermoplasmatota	Methanomassiliicoccaceae	*Methanomassiliicoccus*	42	28	57

**Table 2:** Bacterial taxa and total OTU reads per genus in June (J), August (A) and October (O) in the littoral sediments of the Rila lakes.

Phylum	Family	Genera	OTU Reads per Genus		
			J	A	O
Pseudomonadota	Beijerinckiaceae	*Methylocystis*	1589	2314	2472
	Methylococcaceae	*Methylocaldum*	71	216	179
		*Methyloparacoccus*	3	16	5
		*Methylomagnum*	0	11	5
	Methylomonadaceae	*Crenothrix*	550	2279	738
		*Methylobacter*	361	1158	373
		*Methyloglobulus*	54	183	12
		*Methylomonus*	1	5	1
		*Methylovulum*	6	16	1
		pLW-20	6	11	0
Verrucomicrobiota	Methylacidiphillaceae		27	110	40
Methylomirabilota	Methylomirabilaceae	Ca. *Methylomirabilis*	162	15	5
		SH765B-TzT-35	365	408	203
		Z114MB74	7	6	4

## Data Availability

The original data presented in the study are openly available in the NCBI Sequence Read Archive (SRA) (https://www.ncbi.nlm.nih.gov/sra, accessed on 6 January 2026) under the BioProject accession number PRJNA1398596.

## Abbreviations

The following abbreviations are used in this manuscript:

AOM	Anaerobic methane oxidation
Bliz	Bliznaka lake
Ca.	Candidatus
DC	Dissolved carbon
DIC	Dissolved inorganic carbon
DOC	Dissolved organic carbon
Dol	Dolnoto lake
DON	Dissolved organic nitrogen
m a.s.l.	Meter above sea level
MGs	Methanogenic microorganisms
MTs	Methanotrophic microorganisms
NGS	Next-generation sequencing
OTUs	Operational taxonomic units
PCoA	Principal coordinate analysis
RDA	Redundancy analysis
Rib	Ribnoto lake
SUVA_254_	Specific ultraviolet absorbance at 254 nm
Tri	Trilistnika lake
